# Genetic polymorphisms in the cyclooxygenase-2 gene, use of nonsteroidal anti-inflammatory drugs, and breast cancer risk

**DOI:** 10.1186/bcr1629

**Published:** 2006-12-20

**Authors:** Jing Shen, Marilie D Gammon, Mary Beth Terry, Susan L Teitelbaum, Alfred I Neugut, Regina M Santella

**Affiliations:** 1 Department of Environmental Health Sciences, Mailman School of Public Health, Columbia University, 630 West 168th Street, New York, New York 10032, USA; 2 Department of Epidemiology, School of Public Health, University of North Carolina, CB#7435 McGavern-Greenberg Hall, Chapel Hill, North Carolina 27599, USA; 3 Department of Epidemiology, Mailman School of Public Health, Columbia University, 722 West 168th Street, New York, New York 10032, USA; 4 Department of Community and Preventive Medicine, Mt. Sinai School of Medicine, One Gustave Levy Place, Box 1043, New York, New York 10029, USA

## Abstract

**Introduction:**

The association between use of nonsteroidal anti-inflammatory drugs (NSAIDs) and breast cancer risk remains unclear. Inconsistencies in previously reported findings may be partly due to differences in expression of cyclooxygenase (COX)-2. We hypothesized that genetic polymorphisms (*COX-2 *.926, *COX-2 *.5209, and *COX-2 *.8473) may reduce overall breast cancer risk or risk for subtypes of breast cancer by modulating the inflammatory response and may interact with aspirin or any NSAID use.

**Methods:**

We conducted a population-based, case-control study in which we genotyped 1,067 breast cancer cases and 1,110 control individuals included in the Long Island Breast Cancer Study Project.

**Results:**

No major effects of the three *COX-2 *variant alleles on breast cancer risk were found. A total of eight distinct haplotypes and 18 diplotypes were observed in the population. Overall, no significant associations between *COX-2 *haplotypes/diplotypes and breast cancer risk were observed. Among women who used aspirin or any NSAID there was little evidence for an interaction with the at-risk *COX-2 *genotypes, with one exception. Among women with hormone receptor positive breast cancer, the reduced risk for any NSAID use was only evident among those who had at least one variant C allele of *COX-2 *.8473 (odds ratio = 0.7, 95% confidence interval = 0.5 to 1.0; *P *for the interaction = 0.02). There was no corresponding interaction for aspirin use, possibly because of limited power.

**Conclusion:**

These data provide modest evidence that the C allele of *COX-2 *.8473 may interact with NSAIDs to reduce risk for hormone receptor positive breast cancer.

## Introduction

In recent years, several relatively large studies have examined the association between nonsteroidal anti-inflammatory drugs (NSAIDs) and breast cancer. A meta-analysis of 14 studies (six prospective studies and eight case-control studies) [1] demonstrated a decrease of 20% in the risk for breast cancer among NSAID users. Harris and coworkers [2] observed an inverse relationship between incidence of breast cancer and intake of NSAIDs in a prospective cohort study, with an approximately 40% decreased risk for breast cancer in regular aspirin users. More recent studies [3,4], including our previous analyses from the Long Island Breast Cancer Study Project [5], demonstrated that use of aspirin (the most commonly used NSAID) is associated with a significant reduction in risk for breast cancer, especially for hormone receptor positive tumors. However, data from several recent cohort studies [6,7] have been less consistent.

The anticancer activity of NSAIDs is believed to be due to inhibition of cyclooxygenase (COX)-2, which is over-expressed in many types of cancer, including breast cancer, and plays a major role in tumorigenesis [8,9]. *COX-2 *encodes one of two COX enzymes that catalyze the synthesis of prostaglandins (PGs) from the dietary fatty acid arachidonic acid [10]. Increased COX-2 levels are associated with increased angiogenesis, increased estrogen synthesis, and reduced apoptosis, all of which may stimulate tumor growth [7]. In breast cancer PGs, particularly PGE_2_, have an organ-site-specific effect of increasing the levels of aromatase, thereby increasing estrogen and progesterone synthesis [10-12]. COX-2 expression has been reported in a significant proportion of preinvasive and invasive breast cancers [10,13], and different frequencies of COX-2 over-expression have been observed in subgroups of breast cancer patients by hormone receptor status (estrogen receptor [ER] and progesterone receptor [PR]). More frequent elevation in COX-2 expression was noted in ER/PR-negative breast cancer [14]. COX-2 expression also had a different impact on prognosis in subgroups of tumors. Elevated expression of COX-2 was associated with poor survival in ER-positive or PR-positive tumors (*P *< 0.002) but not in ER/PR-negative ones [14]. Therefore, it is biologically plausible that the protective effect of NSAIDs comes, at least in part, from limiting either the amount or activity of COX-2 present in the cell, both of which are partly determined by specific polymorphisms [15-18].

The previous inconsistent findings on the association between NSAIDs and breast cancer risk might be explained by interindividual differences in *COX-2 *gene expression or be limited to certain subtypes of breast cancer. Thus, we hypothesized that potential functional genetic polymorphisms (variant alleles or haplotypes/diplotypes) in *COX-2 *that result in altered expression and/or activity of the protein may modulate the inflammatory response, modifying overall breast cancer risk or risk for subtypes of breast cancer. Single nucleotide polymorphisms (SNPs) in the promoter and 3'-untranslated region (UTR) of *COX-2 *were previously reported to modulate the risks for prostate, colorectal, esophageal, gastric, bladder, biliary tract, and non-small-cell lung cancer [17,19-31]. A few previous reports showed that the *COX-2 *.926 G→C polymorphism examined here can result in higher COX-2 expression and lead to an approximate 30% reduction in promoter activity *in vitro *[15,18]. No functional studies of other *COX-2 *polymorphisms potentially associated with cancer risks have been reported. Little is known regarding *COX-2 *polymorphism and breast cancer risk and its potential interactions with aspirin and NSAID use [32-35].

## Materials and methods

### Study population

The study population of the Long Island Breast Cancer Study Project has been described in detail previously [36]. In brief, cases were adult female residents of Nassau and Suffolk counties on Long Island, New York, who were of any age or race, spoke English, and were newly diagnosed with *in situ *or invasive breast cancer between 1 August 1996 and 31 July 1997. Control individuals were frequency matched to the expected age distribution of the cases and were identified through random digit dialing for women aged under 65 years and through the Center for Medicare and Medicaid Services rosters for women aged 65 years and older. Eligible control individuals were women who spoke English and who resided in the same Long Island counties as the cases, but who had no personal history of breast cancer. The study was conducted with approval from participating institutional review boards, and in accordance with an assurance filed with and approved by the US Department of Health and Human Services.

Interview response rates among eligible cases and controls were 82.1% (*n *= 1,508) and 62.8% (*n *= 1,556), respectively. Of those who completed the 100-minute main questionnaire, 73.1% of cases (1,102) and 73.3% of controls (1,141) donated a blood sample [36]. As previously reported, an increase in breast cancer among women on Long Island was found to be associated with lower parity, late age at first birth, little or no breastfeeding, a family history of breast cancer, and increasing income and education [36]. Results were similar when the analyses were restricted to respondents who donated blood [36] or to those with DNA available for these analyses (data not shown). Factors that were found to be associated with decreased likelihood that a respondent would donate blood included increasing age, past smoking status, ever consuming alcohol, ever breastfeeding, ever using hormone replacement therapy, ever using oral contraceptives, and ever having had a mammogram. Case-control status was not a predictor of blood donation [36].

### Selection of single nucleotide polymorphisms

The human *COX-2 *gene contains 10 exons and spans 8.3 kilobases [37]. We collected data on *COX-2 *polymorphisms from publicly available databases, such as dbSNP [38], UCSC Genome Bioinformatics [39], and GeneCards [40]. A total of 124 SNPs in *COX-2 *were identified and the studied SNPs chosen according to the following criteria: (a) a minor allele frequency of 5% or greater in Caucasians, according to literature data; and (b) more than 80% homology between human and rat/mouse genome, or (c) previous laboratory evidence indicating a significant functional effect, or (d) previous epidemiologic findings indicating associations with cancer susceptibility. Finally, three SNPs were selected and genotyped for the present study: *COX-2 *.926 G→C (promoter region, rs20417), which satisfies all four criteria; and *COX-2 *.5209 G→T (intron 5, rs20432) and *COX-2 *.8473 C→T (exon 10, 3'-UTR, rs5275), both of which satisfy three of the four criteria (specifically, criteria a, b, and d).

### Genotyping

Genomic DNA was extracted [41] by standard RNase/proteinase K and phenol/chloroform treatment and genotyped by a fluorescence polarization method, using a commercial AcycloPrime™-FP SNP Detection Kit obtained from PerkinElmer Life Sciences (Boston, MA, USA) [42]. The forward and reverse primers were designed according to the human *COX-2 *gene sequence (GeneBank accession number D28235.1) and were (respectively) as follows: 5'-CAT TTA GCG TCC CTG CAA AT-3' and 5'-TAC CTT CAC CCC CTC CTT GT-3' for *COX-2 *.926; 5'-CAT GAT TAT GCC GCT TTC AA-3' and 5'-TCC ACC AAA GCT ACA AAC TGA-3' for *COX-2 *.5209; and 5'-TTC CAA TGC ATC TTC CAT GA-3' and 5'-TCA AAC AAG CTT TTA CAG GTG A-3' for *COX-2 *.8473. The sequences of TDI (Template-directed Dye-terminator Incorporation) probes were designed using Primer 3 software [43] as follows: forward 5'-ATT ATG AGG AGA ATT TAC CTT TCC C-3' for *COX-2 *.926; reverse 5'-ACT TCA CTA TGA TGA TAT GGT AAT T-3' for *COX-2 *.5209; and reverse 5'-ATT TTT CTG TCA TCA AAC AAA AAC A-3' for *COX-2 *.8473. The three allele-specific dye terminators used for each of the three SNPs were G/C, C/A and G/A, respectively. The assay was validated by sequencing individuals with all three genotypes, and these known samples were used as positive controls on each plate.

The laboratory staff was blind to the case/control status of individuals. Based on 272 duplicated quality control samples randomly included in genotyping, an estimated error rate of 0.6% (5/816) was observed. Genotyping data were available for 1,067 (96.8%) cases and 1,110 (97.3%) controls who donated blood, which represent 70.8% (1,067/1,508) of eligible cases and 71.3% (1,110/1,556) of eligible controls.

### Questionnaire data

As part of the interviewer-administered main questionnaire, women were asked to report their intake of aspirin, ibuprofen, and acetaminophen. For 1,028 (96.3%) cases and 1,029 (92.7%) controls, both questionnaire data and DNA for genotyping were available. As previously described [5], 'ever use' was defined as taking aspirin, ibuprofen, and/or acetaminophen at least once a week for 6 months or longer. Ever use of any one of these drugs was defined as 'ever NSAID user'. Other factors considered to be potential confounders (see below) were also assessed as part of the in-person interview [36].

### Statistical analysis

Hardy-Weinberg equilibrium was tested to compare the observed and expected genotype frequencies among cases and controls, respectively [44]. Bivariate analyses were conducted to compare distributions of covariates among cases and controls. Unconditional logistic regression with SAS version 9.0 (SAS Institute, Inc., Cary, NC, USA) was used to estimate odds ratios (ORs) and corresponding 95% confidence intervals (CIs), adjusting for potential confounding factors [45]. The potential confounding variables were assessed individually by comparing the log likelihood ratios derived from a model with and without the variable [45]. Factors that changed the OR estimation of the main effects of genotyping by more than 10% were considered to be potential confounders; however, none of the covariates considered fulfilled this criterion. Variables found not to confound the associations of interest included the following: age at menarche, parity, lactation, months of lactation, age at first birth, number of miscarriages, history of fertility problems, alcohol drinking, race, education, religion, and marital status. All models were therefore adjusted only for the frequency matching factor age at reference, by 5-year age group (defined as age at diagnosis for cases and age at identification for controls).

Haplotype and diplotype frequencies were estimated from genotype data by PHASE version 2.1.1 [46,47], based on Bayesian algorithm. Haplotypes and diplotypes were selected according to the corresponding occurring probabilities with a higher likelihood (with >0.95 as the cut-point) [46,47]. The distribution of haplotypes in the cases and controls was compared by χ^2 ^test [48]. The most common haplotype GTT was selected as the reference in the analysis. The risk for breast cancer was estimated for each diplotype compared with the reference (GTT/GTT), with adjustment for age at reference. Diplotypes were treated as categorical variables and were incorporated as dummy variables in the logistic regression models.

Gene-environment interactions were tested by evaluating departures from multiplicative interaction models by including main effect variables and multiplicative interaction terms in the logistic regression model with Wald statistic [45]. Because of our prior data on the effects of NSAIDs by hormone receptor status, polytomous logistic regression was used to estimate the ORs for breast cancer, with the cases categorized by joint hormone receptor status (ER and PR) of the tumor [45].

## Results

The distributions of *COX-2 *.5209 and *COX-2 *.8473 genotypes were compatible with those expected from Hardy-Weinberg equilibrium, and the frequencies of the variant G and C alleles were respectively 20.0% and 34.1%. The genotype distribution for *COX-2 *.926 significantly deviated from Hardy-Weinberg equilibrium in both controls and combined cases and controls (both *P *values below 0.003).

No main effects on reduced breast cancer risk were found for the three *COX-2 *SNPs, even when women were categorized by menopausal status (Table 1). The frequency of carrying at least one variant allele (*C *allele in *COX-2 *.926, *G *allele in *COX-2 *.5209, or *C *allele in *COX-2 *.8473) did not differ significantly from the controls, even when breast cancer groups were stratified based on hormone receptor status (data not shown).

Haplotypes and diplotypes for the three *COX-2 *SNPs reconstructed using PHASE software [46] resulted in eight distinct haplotypes and 18 diplotypes. Five haplotypes and eight diplotypes were sufficiently frequent to calculate ORs (Table 2). The three common haplotypes (GTT, CGC, and GTC) accounted for more than 94.7% of the alleles. None of the haplotypes exhibited an association with breast cancer risk in this population. The three most frequent diplotypes were GTT/GTT, GTT/CGC, and GTT/GTC. Compared with the most prevalent reference diplotype (GTT/GTT), an uncommon diplotype (GTT/GGC) was associated with a 50% decreased risk for breast cancer (adjusted OR = 0.5, 95% CI = 0.2 to 0.9). The OR remained unchanged when the diplotype was compared with all other diplotype combinations. However, this result is based on very small numbers of individuals. No strong associations were observed between other diplotypes and breast cancer risk.

An inverse association between ever use of aspirin and breast cancer risk was previously reported in this population [5], and a similar result was observed when the analyses were restricted to women who donated blood (OR = 0.80, 95% CI = 0.65 to 0.98). When we evaluated the associations between ever use of aspirin and breast cancer risk stratified by *COX-2 *genotype, the decreased OR observed among ever users of aspirin who carried at least one variant C allele of *COX-2 *.8473 (OR = 0.7, 95% CI = 0.5 to 0.9) compared with nonusers carrying the wild-type TT genotype (Table 3) was similar to the overall OR obtained from ever users of aspirin. No significant interaction was observed (*P *for interaction = 0.77). When restricted to hormone receptor positive cases, the reduction in breast cancer risk was slightly stronger for carriers of at least one variant C allele of *COX-2 *.8473 who ever used aspirin (OR = 0.6, 95% CI = 0.4 to 0.9; Table 4), although the modest heterogeneity in the ORs was not statistically significant. However, there was significant heterogeneity in the ORs for the interaction between ever NSAID use (including both aspirin and nonaspirin NSAID use) and carrying variant C allele of *COX-2 *.8473 (OR = 0.7, 95% CI = 0.5 to 1.0; *P *for interaction = 0.02). No significant interaction between ever use of aspirin, ever use of NSAIDs, and variant C allele of *COX-2 *.8473 was observed in ER/PR-negative breast cancer (Table 4).

When analyzed at the diplotype level, the ORs of ever use of any NSAID alone and carrying the GTT/GGC diplotype alone were, respectively, 0.9 (95% CI = 0.8 to 1.1) and 0.6 (95% CI = 0.2 to 1.4) compared with nonusers carrying all other diplotypes (data not shown). Ever use of any NSAID was associated with a pronounced reduction in OR among women carrying the diplotype of GTT/GGC (OR = 0.3, 95% CI = 0.1 to 0.9) compared with nonusers with all other diplotypes, but numbers were small in these groups and no significant interaction was observed (data not shown).

## Discussion

The COX-2 pathway is now recognized to be important in human cancer development and progression [49]. Numerous studies have suggested a role for *COX-2 *in the initiation, promotion, and progression of cancers in different organs [50-52]. However, little is known about the role of sequence variation within *COX-2 *in breast cancer, and modification with NSAID use [32-35]. In the present study, no overall associations between the three studied *COX-2 *variant alleles and breast cancer risk were found. This finding is consistent with an observation from a nested case-control study conducted in Danish women (361 breast cancer cases and 361 matched controls) [35], but it is not consistent with a report that indicated that the homozygous *COX-2 *. 8473-CC genotype was associated with breast cancer risk (OR = 2.1, 95% CI = 1.3 to 3.3) in an Austrian population (500 cases and 500 controls) [33]. The frequencies of variant CT and CC genotypes in our cases were similar to those in the prior studies [33,35]. We found little evidence to suggest that intake of aspirin or ever NSAID use interacted with *COX-2 *genotypes to affect overall breast cancer risk. However, among women with hormone receptor positive breast cancer, there was some evidence to suggest that the reduction in risk associated with ever NSAID use (including both aspirin and nonaspirin NSAID use) was limited to women who carried the variant C allele for *COX-2 *.8473; however, there was no corresponding interaction between this polymorphism and ever aspirin use alone in this subgroup.

Several previous studies, including the SNP500 database [53], have reported the frequency of *COX-2 *.8473 variant C allele in the 3'-UTR in Caucasian populations to range between 31.4% and 46.5% [19-21], which is consistent with our finding (34.1%). Our larger sample size results in a more stable genotyping frequency compared with prior small studies. Although a small previous study [20] found that carriers of the *COX-2 *.8473 variant C allele had a significantly increased risk for lung cancer (ORs 2.12 for CT genotype and 4.28 for CC genotype), an expanded study in the same population [28] failed to reproduce the association (ORs 0.96 for CT genotype and 0.97 for CC genotype). Furthermore, the significantly reduced OR observed for the GTT/GGC diplotype (OR = 0.5, 95% CI = 0.2 to 0.9) in the present study is probably a false-positive finding caused by chance because of the small number of observations (34 carriers of the diplotype with NSAID data).

Our findings on the roles of *COX-2 *.8473 polymorphism and NSAIDs among women with hormone receptor positive breast cancer are biologically plausible. Polymorphisms present in the regulatory 3'-UTR of *COX-2 *might hinder the binding of RNA-binding proteins and modulate mRNA stability and degradation, and finally decrease the production of protein [54-56]. Therefore, it is reasonable to speculate that the *COX-2 *.8473 polymorphism, located downstream of the stop codon in the adenylate/uridylate-rich 3'-UTR region [20], could partly decrease mRNA stability and expression either by modifying the efficiency of polyadenylation signals or by affecting the binding affinity of regulatory elements. This could result in decreased cellular COX-2 activity and reduced inflammatory response, angiogenesis, and tumor growth. Continuous use of any NSAIDs appears to result, via a local blockade of the COX-2 enzyme, in a reduction in PGE_2 _and aromatase synthesis. The consequent reduction in the local production of estrogen, in turn, is associated with the reduced breast cancer risk [10-13,57,58]. However, the fact that there is no direct laboratory evidence on the function of the *COX-2 *.8473 polymorphism may increase the likelihood of a spurious association or indicate the presence of some other functional SNPs in strong linkage disequilibrium with this SNP.

The more pronounced interaction between the *COX-2 *.8473 polymorphism and ever NSAID use in ER-positive or PR-positive tumors than that in ER/PR-negative tumors is of particular interest. This might be an indication that different mechanisms are involved in subtypes of breast cancer. The lower frequency of COX-2 over-expression in hormone receptor positive tumors may make it easier to observe a significant protective effect of NSAID use among carriers of the variant C allele of *COX-2 *.8473. This is consistent with the observation that elevated expression of COX-2 was statistically significantly associated with poor survival in ER-positive or PR-positive tumors but not in ER/PR-negative ones [14]. The same dose of NSAIDs might have a protective effect in ER-positive or PR-positive tumors by acting on an inflammation-related pathway through decreasing COX-2 activity, reducing PGE_2 _and aromatase synthesis, and decreasing estrogen production. However, there are also several discrepant reports showing that long-term daily use of aspirin significantly increased risk for ER/PR-negative breast cancer [7], and ER-negative tumors are more sensitive to chemotherapy than are ER-positive ones [59]. We did not observe a significant interaction between ever aspirin use, ever NSAID use, and the variant C allele of *COX-2 *.8473 in ER/PR-negative breast cancer, perhaps because of limited power (Table 4). The nonsignificant interaction observed in ever aspirin use might be a reflection of either inadequate power or lack of a true association. Considering the biologic mechanism of action of aspirin and nonaspirin NSAIDs and the results obtained in the present study (Table 4), we believe that this inconsistency is due to limited power resulting from the small sample size in some subgroups of aspirin users. The observed significant interaction between ever NSAID use and the variant C allele of *COX-2 *.8473 for decreasing breast cancer risk also included, to a certain extent, a contribution of ever aspirin use. The ORs for interactions between the genotype and either ever aspirin use or ever NSAID use were essentially similar (OR 0.6, 95% CI = 0.4 to 0.9 versus 0.7, 95% CI = 0.5 to 1.0). This is consistent with the results from our analysis of the interactions between nonaspirin NSAID use alone, aspirin use alone, and *COX-2 *.8473 polymorphism (data not shown). Thus, the difference in interaction between ever aspirin use, ever NSAID use, and the *COX-2 *.8473 polymorphism might well be the result of limited power for subgroup analyses rather than lack of a true association.

The relatively large sample size, population-based study design, and availability of both genotyping and NSAID information are the strengths of the study. The NSAID data were obtained from retrospective reporting of medication use, which might lead to recall bias. However, in order to explain our findings, cases should under-report more than controls or the accuracy of reporting should be related to genotype status or ER/PR status. Thus, it is unlikely that recall bias played a major role in explaining the present findings. Previous sensitivity analysis also indicated that the missing data would not have altered the overall conclusion of a protective effect between aspirin use and breast cancer [5].

Another consideration was that our NSAIDs information did not include the use of prescription drugs containing NSAIDs (such as naproxen, etodolac, ketoprofen, and sulindac). The presumed 'unexposed' women who used prescription NSAIDs would be included in the nonuser category and lead to an underestimation of inverse associations. Such a misclassification of users as nonusers would tend to bias estimates of NSAID effects toward the null. The extensive information collected in the study for evaluating confounding factors and effect modifiers allowed us to assess confounding in the data analysis. ORs changed less than 10% when considering potential confounding factors individually or in multivariate models, indicating that the effects of confounding are limited. Inclusion of age at reference in logistic models minimized any possible residual confounding effect of age. Although our questionnaire also included variables on duration and frequency of aspirin and other NSAID use, providing some information on dose, the small sample size in some subgroups when categorized by *COX-2 *genotype limited power to evaluate dose-response effects.

## Conclusion

This population-based case-control study provided some evidence to support our hypothesis that the *COX-2 *.8473 variant C allele may be a genetic modifier for ever use of NSAIDs and reduced breast cancer risk among hormone receptor positive cases, but we observed no corresponding interaction with aspirin use alone, which may be due to reduced power in this subgroup. Because there has been no laboratory evaluation of the biologic function of *COX-2*.8473 polymorphism, additional studies to examine the functionality of the polymorphism and the potential interactions with various inflammation-related genes are warranted.

## Abbreviations

CI = confidence interval; COX = cyclooxygenase; ER = estrogen receptor; NSAID = nonsteroidal anti-inflammatory drug; OR = odds ratio; PG = prostaglandin; PR = progesterone receptor; SNP = single nucleotide polymorphism; UTR = untranslated region.

## Competing interests

The authors declare that they have no competing interests.

## Authors' contributions

JS was responsible for genotyping polymorphisms, data analyses and interpretation, and drafting of the manuscript. MG contributed to study design, sample collection and manuscript preparation, and provided expertise in data analyses. MBT, SL, and AN contributed to the study design, sample collection, and manuscript preparation. RS was responsible for supervising laboratory work, and contributed to study design, biospecimen processing and manuscript preparation. All authors read and approved the final manuscript.

## Figures and Tables

**Table 1:** *COX-2 *genotypes and risk for breast cancer by menopausal status: Long Island Breast Cancer Study Project, 1996 to 1997

Genotypes	Cases (*n *)^a^	Controls (*n *)^a^	Crude OR (95% CI)	Adjusted OR (95% CI)^b^
All women				
*COX-2 *.926 (rs20417)				
GG	670	691	1.0 (ref.)	1.0 (ref.)
GC + CC	387	414	1.0 (0.8–1.1)	1.0 (0.8–1.2)
*COX-2 *.5209 (rs20432)				
TT	685	694	1.0 (ref.)	1.0 (ref.)
TG + GG	372	399	0.9 (0.8–1.1)	0.9 (0.8–1.1)
*COX-2 *.8473 (rs5275)				
TT	475	467	1.0 (ref.)	1.0 (ref.)
TC + CC	585	635	0.9 (0.8–1.1)	0.9 (0.8–1.1)
Premenopausal women				
*COX-2 *.926 (rs20417)				
GG	217	234	1.0 (ref.)	1.0 (ref.)
GC + CC	124	142	0.9 (0.7–1.3)	1.0 (0.7–1.4)
*COX-2 *.5209 (rs20432)				
TT	225	229	1.0 (ref.)	1.0 (ref.)
TG + GG	116	142	0.8 (0.6–1.1)	0.9 (0.6–1.2)
*COX-2 *.8473 (rs5275)				
TT	148	153	1.0 (ref.)	1.0 (ref.)
TC + CC	193	223	0.9 (0.7–1.2)	0.9 (0.7–1.2)
Postmenopausal women				
*COX-2 *.926 (rs20417)				
GG	438	428	1.0 (ref.)	1.0 (ref.)
GC + CC	253	255	1.0 (0.8–1.2)	1.0 (0.8–1.2)
*COX-2 *.5209 (rs20432)				
TT	445	438	1.0 (ref.)	1.0 (ref.)
TG + GG	246	238	1.0 (0.8–1.3)	1.0 (0.8–1.3)
*COX-2 *.8473 (rs5275)				
TT	318	295	1.0 (ref.)	1.0 (ref.)
TC + CC	376	385	0.9 (0.7–1.1)	0.9 (0.7–1.1)

^a^*n *values for cases and controls among all women are not equal to the sum of premenopausal and postmenopausal women because of missing data for menopausal status. ^b^Adjusted for age at reference. CI, confidence interval; OR, odds ratio.

**Table 2:** *COX-2 *haplotype and risk for breast cancer: Long Island Breast Cancer Study Project, 1996 to 1997

*COX-2 *haplotype/diplotype status	Cases (*n *)	Controls (*n *)	Adjusted OR (95% CI)^a^	*P *value
Haplotype^b^				
GTT	1,359	1,405	1.0 (ref.)	
CGC	363	388	1.0 (0.8–1.2)	0.85
GTC	299	306	1.0 (0.8–1.2)	0.95
GGC	38	60	0.7 (0.5–1.0)	0.06
CTT	27	33	0.8 (0.5–1.4)	0.48
Diplotype (haplotype pairs)				
GTT GTT	434	432	1.0 (ref.)	
GTT CGC	239	262	0.9 (0.7–1.1)	0.45
GTT GTC	186	194	1.0 (0.7–1.2)	0.69
GTC CGC	49	51	0.9 (0.6–1.4)	0.79
GTC GTC	26	25	1.0 (0.6–1.8)	0.94
GTT GGC	12	27	0.5 (0.2–0.9)^c^	0.03
GTT CTT	19	24	0.7 (0.4–1.4)	0.36
CGC CGC	25	23	1.1 (0.6–2.1)	0.68
Others	47	36	1.3 (0.8–2.0)	0.31

^a^Adjusted for age at reference. ^b^*COX-2 *haplotype composed of three polymorphic sites: *COX-2.926 *G→C, *COX-2.5209 *T→G, and *COX-2.8473 *T→C. ^c^The comparison with all other diplotype combinations was also statistically significant (OR = 0.5, 95% CI = 0.2 to 0.9; *P *= 0.03). CI, confidence interval; OR, odds ratio.

**Table 3:** *COX-2 *genotypes, NSAIDs use, and risk for breast cancer: Long Island Breast Cancer Study Project, 1996 to 1997

	No use		Ever use	
	
	Cases/controls	OR (95% CI)^a^	Cases/controls	OR (95% CI)^a^
Aspirin				
*COX-2 *.926 (rs20417)				
GG	511/484	1.0 (ref.)	138/156	0.8 (0.6–1.0)
GC + CC	285/291	0.9 (0.8–1.1)	84/93	0.8 (0.6–1.1)
*COX-2 *.5209 (rs20432)				
TT	521/485	1.0 (ref.)	145/160	0.8 (0.6–1.0)
TG + GG	275/282	0.9 (0.7–1.1)	78/86	0.8 (0.6–1.1)
*COX-2 *.8473 (rs5275)				
TT	363/332	1.0 (ref.)	98/101	0.8 (0.6–1.1)
TC + CC	436/439	0.9 (0.8–1.1)	124/148	0.7 (0.5–0.9)^b^
Any NSAID				
*COX-2 *.926 (rs20417)				
GG	418/412	1.0 (ref.)	235/240	0.9 (0.7–1.1)
GC + CC	245/249	0.9 (0.8–1.2)	126/137	0.8 (0.6–1.1)
*COX-2 *.5209 (rs20432)				
TT	427/417	1.0 (ref.)	243/240	0.9 (0.7–1.1)
TG + GG	237/237	1.0 (0.8–1.2)	118/133	0.8 (0.6–1.1)
*COX-2 *.8473 (rs5275)				
TT	290/285	1.0 (ref.)	175/155	1.1 (0.8–1.4)
TC + CC	376/371	1.0 (0.8–1.3)	186/223	0.8 (0.6–1.0)

^a^Adjusted for age at reference. ^b^Multiplicative interaction term was not significant (*P *for interaction = 0.69), by Wald statistic. CI, confidence interval; OR, odds ratio.

**Table 4:** *COX-2 *.8473 genotype, NSAIDs use, and risk for breast cancer by hormone receptor status, Long Island Breast Cancer Study Project, 1996–1997

*COX-2 *.8473 genotype	Aspirin/any NSAID	No use		Ever use	
		
		Cases/controls	OR (95% CI)^a^	Cases/controls	OR (95% CI)^a^
ER or PR positive					
TT	Aspirin	191/332	1.0 (ref.)	46/101	0.7 (0.5–1.0)
TC + CC		237/439	1.0 (0.7–1.2)	61/148	0.6 (0.4–0.9)^b^
TT	Any NSAID	147/285	1.0 (ref.)	92/155	1.1 (0.8–1.5)
TC + CC		210/371	1.1 (0.9–1.5)	89/223	0.7 (0.5–1.0)^c^
ER and PR negative					
TT	Aspirin	44/332	1.0 (ref.)	13/101	1.0 (0.5–1.8)
TC + CC		53/439	0.9 (0.6–1.4)	22/148	1.1 (0.6–1.9)^d^
TT	Any NSAID	40/285	1.0 (ref.)	18/155	0.8 (0.5–1.5)
TC + CC		39/371	0.8 (0.5–1.2)	37/223	1.2 (0.7–1.9)^e^

^a^Adjusted for age at reference. ^b^Multiplicative interaction term was not significant (*P *for interaction = 0.78) by Wald statistic. ^c^Multiplicative interaction term was significant (*P *for interaction = 0.02) by Wald statistic. ^d^Multiplicative interaction term was not significant (*P *for interaction = 0.59) by Wald statistic. ^e^Multiplicative interaction term was not significant (*P *for interaction = 0.10) by Wald statistic. CI, confidence interval; ER, estrogen receptor; NSAID, nonsteroidal anti-inflammatory drug; OR, odds ratio; PR, progesteron receptor.
